# Tools for Controlling Activity of Neural Circuits Can Boost Gastrointestinal Research

**DOI:** 10.3389/fphar.2016.00043

**Published:** 2016-03-04

**Authors:** Gabriella Aviello, Giuseppe D'Agostino

**Affiliations:** ^1^National Children's Research Centre, Our Lady's Children's HospitalDublin, Ireland; ^2^Rowett Institute of Nutrition and Health and Institute of Medical Sciences, University of AberdeenAberdeen, UK

**Keywords:** intestinal permeability, inflammatory bowel diseases, microbiota-gut-brain axis, DREADD, chemogenetics, optogenetics

Compelling evidence indicates that alterations of intestinal homeostasis, such as “leaky” epithelial barrier and changes in microbiota composition, can be associated with pathological adaptations of brain functions (Carabotti et al., 2015). On the other hand, just as disrupted gut homeostasis affects the brain, the brain can also exert a profound influence on the intestine as indicated by the elicitation of inflammatory bowel disease (IBD) or IBD-like conditions following stress and depression (for review see Di Giovangiulio et al., 2015). Recently, the recognition that microbiota influences signaling pathways regulating the central nervous system (CNS) has led to the concept of “microbiota–gut–brain axis” (Cryan and Dinan, 2012). Indeed, the routes of communication between brain and intestinal microbes rely on the immune system activation, and on the capability of microbiota to produce a number of neurochemicals (GABA, serotonin, dopamine) regulating learning, memory, and mood (Dinan et al., 2015; Moloney et al., 2015).

With the evolving concept of psychoneuroimmunology the modalities by which the brain can influence intestinal functions and *vice versa* are becoming more evident, although mechanistically this bidirectional communication remains ill defined. For instance, patients with quiescent IBD show an increased probability of relapse when encountering chronic stress, adverse life events and depression (Mawdsley and Rampton, 2005). Similarly, the induction of an experimental depressive-like state induced by olfactory bulbectomy in mice caused reactivation of colitis (Ghia et al., 2009), suggesting that properly functioning central neuronal circuits are crucial for the maintenance of gut homeostasis. On the other hand, changes of gut microbiota composition can directly affect brain development in growing infants (Douglas-Escobar et al., 2013) and discrete perturbations of intestinal microflora were shown to induce behavioral abnormalities in mice (Desbonnet et al., 2015).

Chronic intestinal inflammation observed in IBD patients is also associated with extra-intestinal symptoms including anxiety and depression-like behaviors (Graff et al., 2009) as well as gastrointestinal morbidities induced by alterations of the autonomic nervous system (ANS; Lindgren et al., 1993). Motility, secretion and vasoregulation are controlled by sympathetic and parasympathetic extrinsic branches, consisting of noradrenergic nerves dynamically interacting with immune cells and enteric neurons (located in the mucosa and the submucosa), and the vagus nerve broadly innervating the intestinal wall up to the myenteric plexus. An increase in brain cholinergic activity attenuates experimental colitis by the initiation of vagal anti-inflammatory pathways in the periphery (Ghia et al., 2006), whereas *via* their efferents to gut-associated lymphoid tissues including Payer's patches and mesenteric lymph nodes, sympathetic fibers directly influence plasma cell, T cell and dendritic cell responses and suppress cytokine secretion and macrophage phagocytosis (Di Giovangiulio et al., 2015).

Despite anatomical, preclinical and clinical evidence, the identity of neurons and the circuitry governing the gut-brain bidirectional communication are still largely unknown. Methodological difficulties associated with unraveling discrete circuits regulating the gut-brain axis have slowed down the achievement of crucial and clinically meaningful information.

Recently new technologies became available that allow unprecedented opportunities for manipulating neurons and circuits. These new tools accelerated neuroscience research over the last 10 years to a vertiginous speed; a step change that has changed both experimental approaches and research questions. Just like any other circuits, neuronal circuits can be now turned on and off, providing the availability of one or more switchers positioned at defined circuit's nodes. For neurons, switchers are (mostly) receptors that have been engineered to be remotely operated. Genetics provides then a means to express such switchers within the circuits. Switchers activated by drugs are collectively defined as chemogenetics (for review see Sternson and Roth, 2014), while switchers activated *via* light sources are named optogenetics (for review see Tye and Deisseroth, 2012; Figures 1A,B).

**Figure 1:**
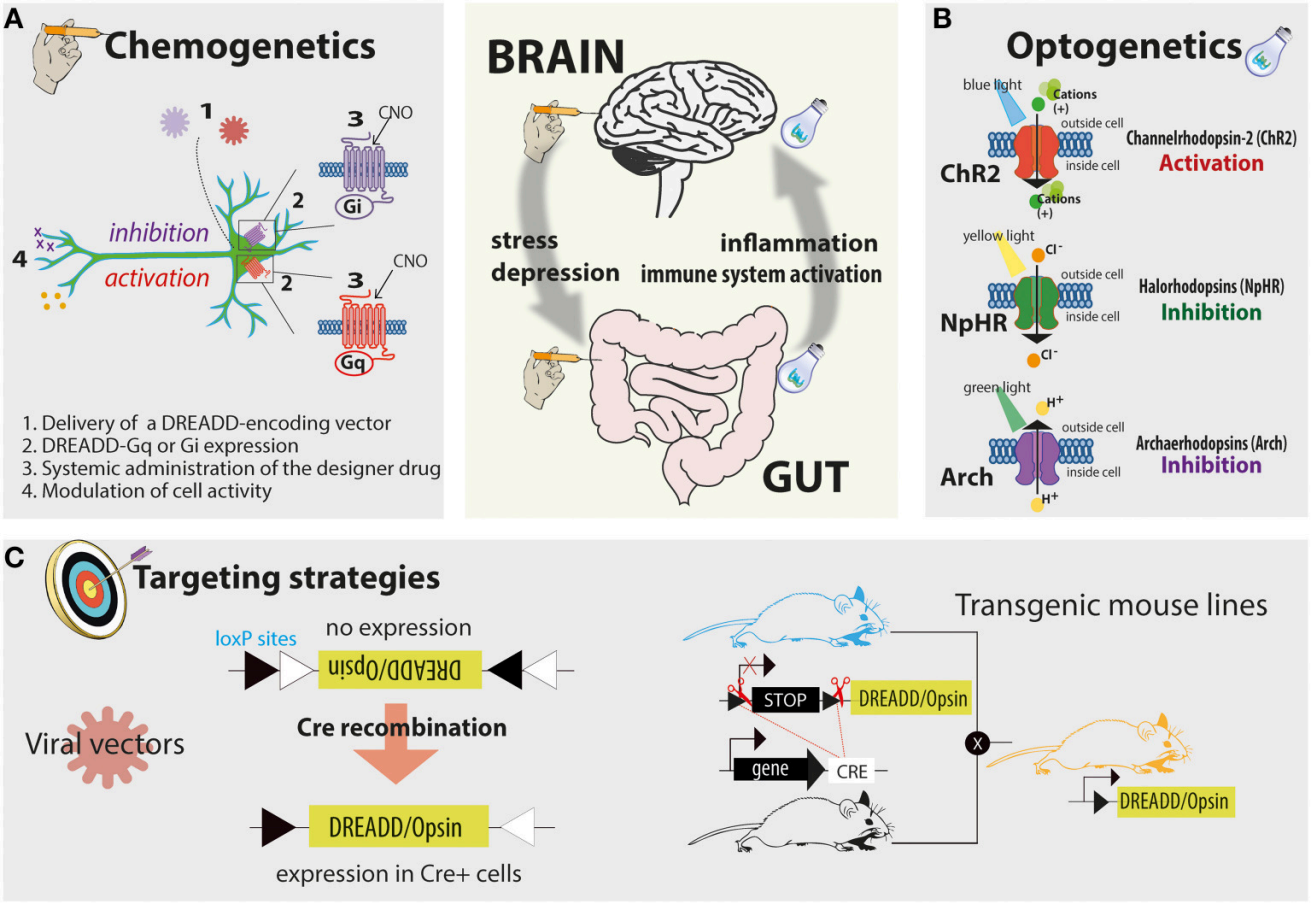
**The proposed tools to study the gut-brain axis include the increasingly popular chemogenetic platform known as DREADD and optogenetics**. In panel **(A)** the principles of the chemogenetic technology are schematized: these include the delivery of a DREADD encoding vector, the expression of the designer receptor in the cell population of interest, and the modulation of this receptor by a designer drug. In panel **(B)** the principles of the optogenetic technology and the mechanisms by which commonly used opsins modulate cell activity are schematized and simplified. In panel **(C)** two examples of cell-specific delivery strategies are reported. Left, in Cre-inducible viral vector the gene of interest is initially positioned in a non-coding orientation; following Cre-mediated recombination the gene of interest is flipped in a coding orientation allowing cell-specific expression. Right, an example of cell-specific targeting by using transgenic mouse lines. When a Cre reporter transgenic mouse line is crossed with a Cre-inducible opsin or DREADD knock-in line cell-specific expression can be achieved.

## Chemogenetics

The chemogenetic platform known as DREADDs (designer receptors exclusively activated by designer drugs) has already shown to be suitable for manipulating neuronal activity in cell types as diverse as glia, pancreatic β-cells, hepatocytes, fibroblasts, and induced pluripotent stem cells. This technology allows the expression of designer receptors whose affinity for endogenous ligands is lost, while affinity for an otherwise inert designer ligand is gained (Figure 1A). For example, DREADDs can be designer muscarinic receptor variants activated by the biologically inert designer drug clozapine-N-oxide (CNO). When CNO binds to DREADD it modulates cellular signaling *via* G protein-coupled receptor cascades. *In vivo* according to the nature of designer receptors employed (Gq-, Gs-, or Gi-coupled), a systemic administration of a designer drug therefore directly modulates the activity of transduced cells (Alexander et al., 2009).

DREADDs can be delivered in rodents *via* viral systems, including adeno-associated viral vectors that express DREADDs only following Cre-mediated recombination, thus facilitating the targeting of genetically defined cell populations (Figure 1C; Krashes et al., 2011).

However, viral systems require localized injections within discrete brain areas or, where possible, localized administrations into the organ of interest—a possible limitation in the context of gastrointestinal manipulations. While it is possible to envision intracolonic inoculation of viral particles, targeting the upper intestinal tract could prove difficult. Nevertheless, knock-in mouse models bearing different DREADD alleles are also available, enabling the expression of DREADD variants within the tissue of interest when crossed with a specific Cre-reporter mouse line (Figure 1C; Alexander et al., 2009).

Chemogenetic tools are not limited to muscarinic-derived DREADD. Designer receptors based on ion channels (Magnus et al., 2011) or an inhibitory designer receptor derived from the κ opioid receptor have also been developed (Vardy et al., 2015); this variety of chemogenetic tools has also the advantage to allow multiplexed approaches. For instance, co-expression of designer receptors activated by different designer drugs allows bidirectional modulation of cell activity within the same experimental subject (Vardy et al., 2015).

The chemogenetic toolbox is continuously growing and today chemogenetics represents undoubtedly an accessible and easy-to-implement technology for *on demand* modulation of cell activity. However, the main limitation of this technology is the lack of temporal control following the administration of a designer drug and the activation of DREADDs, with both onset and duration of the elicited “physiological” phenomena being mainly dictated by the pharmacokinetic features of the designer drug used.

## Optogenetics

Cell activity can also be modulated *via* optogenetic technologies that use light sources to control living tissues, genetically engineered to express light-sensitive proteins (opsins). The most common and best characterized opsins used in optogenetics are: (i) channelrhodopsin 2 (ChR2), able to elicit action potentials time locked to blue light pulses; (ii) halorhodopsin, a yellow light-gated ion pump that moves chloride ions into the cell; and (iii) archaerhodopsin, a green light-driven proton pump that moves protons out of the cell. Halorhodopsin and archaerhodopsin variants have the net effect of silencing active neurons by enabling hyperpolarization of membranes and reducing the likelihood of action potential generation (Figure 1B). Although technically more challenging compared to chemogenetics, this approach offers at least three main advantages: (i) the temporal control over the modulation of cell activity; (ii) the definition of firing patterns with millisecond resolution (owing to the possibility of rapidly modulating the light pulse); and (iii) the manipulation of specific projection sites within the CNS or, notably, in the proximity of innervated organs (due to the capacity of opsins to diffuse along axons and localize at synaptic terminals). The latter is one of the most attractive features of this technique. Indeed, viral transduction strategies enable anterograde-like targeting capabilities, where opsins are expressed in local cell bodies and trafficked to downstream terminals; here, opsin-expressing projections can then be illuminated to control cells by virtue of their efferent connectivity. A proof of concept regarding how optogenetics could be useful in studying the neuronal control of peripheral organ function was recently published. Zeng et al. showed that local optogenetic stimulation of sympathetic inputs at neuro-adipose junctions promotes a local lipolytic response with consequent depletion of the mouse white adipose mass (Zeng et al., 2015).

Although for routine application of optogenetic it is still necessary tethering the experimental subject to lasers or LED light sources, ongoing lines of research also focus on the implementation of portable and implantable micro devices that can be carried by animals during enacted behavior (Kim et al., 2013; Montgomery et al., 2015; Park et al., 2015). While implementation and easy-access to such devices will be a major advance for the neuroscience field as a whole (for review see Kale et al., 2015), implantable and wireless devices will be crucial for a full incorporation of optogenetics into the gastrointestinal research.

## Other approaches

While chemo- and opto-genetic approaches enable *on demand* control of neuronal activity, these technologies (like pharmacological agents) can modulate cell activity in a “non-physiological” manner—this caveat is particularly true when activation strategies are used. Despite the availability of reversible inhibitory chemo- and opto-genetic tools, long-term and cell-specific loss of function approaches are often crucial to test the physiological meaning of a given system (i.e., appearance of phenotype). In this respect, a number of approaches allow permanent inactivation of cell functions. Specific subset of neurons can be genetically manipulated to express the diphtheria toxin (DT) receptor. DT-expressing neurons are ablated by systemically administering DT (Saito et al., 2001). With this approach the experimenter maintains control over the onset of the ablation, an advantage when the role of a given neuronal population has to be interrogated at a specific developmental or disease stage. Similarly, a viral system to promote death of discrete cell populations by expressing a Cre-inducible form of caspase-3 was recently developed (Morgan et al., 2014). Likewise, the expression of an allele encoding the tetanus toxin light chain in genetically defined neuronal populations has proven effective in blocking vesicle release in different experimental conditions (Kim et al., 2009; Xu and Südhof, 2013). Tetanus toxin-based approaches would bypass the need of inducing cell death, thus possibly minimizing the potentially detrimental effect that neuronal death may cause on surrounding non-targeted cells, although confirming that the loss of secretory activity can be difficult.

Besides efferent and afferent activity of the ANS, neuronal regulation of gut homeostasis also originates locally within the intestine, through its own independent nervous system, namely enteric nervous system (ENS): an intricate network of over 100 million neurons grouped in the Auerbach and the Meissner plexi. Enteric fibers projecting to Peyer's Patches and in close vicinity of immune cells control inflammation by means of neurotransmitters, neuropeptides or other signaling molecules (Di Giovangiulio et al., 2015). Thus, tools developed for the manipulation of central circuits will be also useful to interrogate discrete local cell populations within the ENS. For instance, intracolonic inoculation of viral vectors expressing chemogenetic or optogenetic tools could be a strategy to target genetically defined enteric neurons by means of Cre reporter transgenic mouse lines or by using specific neuronal subtype promoters (Figure 1C). Likewise, transgenic mouse lines harnessed to express chemogenetic and optogenetic receptors will provide an invaluable source of intestinal tissue amenable to evaluate *ex vivo* either secretory or contractile activity.

## Conclusions

Owing to their relatively recent development, technologies for neuronal circuit manipulation have been used to interrogate brain networks underpinning behavior and the brain's processing of sensory information. However, the first evidence of their usefulness within the gastrointestinal tract has been recently reported. For instance, a chemogenetic mouse line expressing DREADDs under transcriptional control of the glial fibrillary acidic protein promoter (GFAP; a marker for astrocytes) was used to study intestinal motility by evoking glial Ca^2+^ responses (McClain et al., 2015).

The selectivity and wide range of manipulations that are now possible with these technologies offer an unprecedented and fascinating opportunity to expand our understanding of the gut-brain axis, the behavioral effects associated with intestinal homeostasis alterations and the gut responses following discrete manipulation of defined neuronal networks.

## Author contributions

All authors listed have made substantial, direct and intellectual contribution to the work, and approved it for publication.

### Conflict of interest statement

The authors declare that the research was conducted in the absence of any commercial or financial relationships that could be construed as a potential conflict of interest.
